# Do we publish what we preach? Analysis of the European Society for Surgery of the Shoulder and Elbow Congress publication rates

**DOI:** 10.1186/s13018-017-0620-1

**Published:** 2017-07-24

**Authors:** J. Miquel, S. Fernández-Muñoz, F. Santana, C. Torrens

**Affiliations:** 1 Orthopaedics & Trauma Department, Consorci Sanitari de l’Anoia, Avinguda Catalunya 11, 08700 Igualada, Spain; 2 Orthopaedics & Trauma Department, Parc de Salut Mar. Barcelona, Passeig Marítim 25–29, 08003 Barcelona, Spain

**Keywords:** Publication rates, SECEC congress, Abstracts, Podium presentation, Poster presentation

## Abstract

**Background:**

Scientific congresses have become the most expedient method to communicate novel findings on any research topic. However, an important question is whether this information will be published in peer-reviewed journals. Our aim was to determine the publication rate of the abstracts presented at the European Society for Surgery of the Shoulder and Elbow Congress and analyze factors that may influence this rate.

**Methods:**

A total of 398 abstracts reported in the Abstract Book from the 2008 European Society for Surgery of the Shoulder and Elbow Congress were examined and categorized by oral and poster presentations, topic, and the number of authors listed. A search in PubMed and Google Scholar for subsequent peer-reviewed publications was performed in September 2015. The time to publication after the meeting had been held; the type of journal and its impact factor at the time to publication were recorded for those abstracts that reached peer-reviewed journal publication.

**Results:**

The overall publication rate for the 2008 European Society for Surgery of the Shoulder and Elbow oral and poster presentations was 45.20% after 7 years. The mean time to publication was 18.53 months, and the mean impact factor value was 2.32. Oral presentations were significantly better represented in journals than posters (64.40 vs. 35.40%, *p* < 0.001). Abstracts with a greater number of authors listed had better publication rates (*p* < 0.001).

**Conclusion:**

Less than half of the oral presentations and posters at the 21st European Society for Surgery of the Shoulder and Elbow Congress were published in peer-reviewed journals. Oral presentations with a higher number of authors had an increased likelihood of being published.

## Background

The main goal for researchers with scientific findings is to share their data with the scientific community. New findings or novel techniques are usually first presented to medical societies at congresses or meetings. However, publication in peer-reviewed journals represents the gold standard for disseminating scientific data across the scientific community, as copious data related to a study cannot be covered in-depth at the podium or in a poster presentation.

Interest in a meeting’s or a society’s publication rate has recently risen, as it represents an indicator of the degree and quality of the scientific society’s activity. The publication rate has become a tool for determining the scientific level of the congress. Several orthopedic and trauma surgery societies have published their congress or meeting publication rates [1–22]. Among shoulder and elbow societies, only the Shoulder and Elbow Sessions of the American Academy of Orthopedic Surgery has published the abstract publication rates of the congress [23]. In 2015, the 26th European Society for Surgery of the Shoulder and Elbow (SECEC) Congress celebrated the most successful congress this society has ever had relative to the number of participants. The participants were from more than 50 countries, and the number of submitted abstracts was equally notable [24]. Therefore, the stature of the SECEC justifies a systematic study of its abstracts. P. Hughes and colleagues presented a poster at the 21st SECEC Congress (2008) compared the publication rates of the abstracts presented at the British Elbow and Shoulder Society to those presented at the SECEC. This report is still the only reference to the SECEC Congress publication rate. Therefore, little is known about the fate of abstracts after presentation in an SECEC Congress.

The purpose of this study was to report the SECEC publication rate in peer-reviewed journals and to analyze the characteristics of the abstracts that achieved peer-reviewed journal publication.

## Methods

Papers reported in the Final Program from the 21st SECEC Congress (2008) were included in this study to determine peer-review process survivorship. The Final Program from 2008 was available online, and the time to expected publication was set at 7 years.

All abstracts were investigated using the PubMed and Google Scholar databases in September 2015 to identify any corresponding published articles in the journals listed in the databases. The search parameters included the first author along with the first broad keyword appearing in the abstract title. When not successful, the search was followed by a search for all subsequent authors using the same parameters before declaring an abstract unpublished. An abstract was considered “published” based on the following criteria: when congress papers in the Final Program and their publication in journals could be matched with the same or a similar title or when a coincidence of authors in a title that referred to the same topic was found. In the case of multiple publications per abstract, the time to publication closest to the congress was used. When a publication occurred before the congress was held, the abstract was categorized as “published” if the title of the paper could be attributed to the original SECEC Congress abstract. All these criteria were previously established to quantify congress publication rates [19].

A total of 398 abstracts reported in the 2008 SECEC Congress Final Program were classified based on several characteristics to determine which of these abstracts had a greater likelihood of being published. The number of authors listed and the time span to publication (in months) were obtained by calculating the time from September 2008 to the month of journal publication. Papers were categorized by topic, including the elbow, proximal humeral fractures, the rotator cuff, degenerative shoulder pathologies/arthroplasties, instability*,* the clavicle and AC joint, miscellaneous/basic science, and tips and tricks/new techniques. The peer-reviewed journal and its impact factor at the time to publication were recorded for those abstracts that achieved peer-reviewed journal publication using the Journal Citation Reports® (Clarivate Analytics, 2017) [25].

The statistical analysis was performed using the SPSS 18.0 package (SPSS Inc., Chicago, IL). A descriptive analysis was initially performed. A non-parametric Wilcoxon test was also performed to compare groups with an asymmetric distribution, while Spearman’s correlation was used to evaluate the associations between groups. A chi-squared test was performed to evaluate the association between dichotomous variables, such as the publication of the abstract before vs. after the congress.

Confidence intervals were estimated at 95% of the estimators, and the differences were considered significant at *p* < 0.05.

## Results

A total of 522 abstracts were submitted for oral or poster presentations in this congress, and 398 were accepted for presentation at the 21st SECEC Congress. Thus, 76.24% of the abstracts submitted were accepted for either oral or poster presentations. Regarding oral presentations, 135 (25.86%) of the total abstracts submitted were accepted while 263 abstracts (50.38%) were accepted as poster presentations.

Of the 398 abstracts accepted for the 21st SECEC Congress, 135 (33.92%) were categorized as podium presentations, while 263 (66.08%) were poster presentations. Abstracts that survived the peer-review process were published in 25 different journals, with a mean impact factor value of 2.32 (0.18–7.33). The mean time span to publication was 18.53 months (−28 months to 77 months). Twenty-five abstracts were published before the SECEC Congress was celebrated, with a mean of 18.5 months (−28 months to 0 months) in advance. The mean number of authors listed on the abstracts was 4.05 (1–7). The classification relative to the topic of the paper is shown in Table 1.

**Table 1:** Distribution of abstracts according to topic, and their publication rate

Abstract topic	Number of abstracts	Percentage of abstracts	Publication rate (%)
Elbow	50	12.6	42
Proximal humeral fractures	42	10.6	33.3
Rotator cuff	69	17.3	39.1
Shoulder degenerative/arthroplasty	38	9.5	55.3
Instability	37	9.3	48.6
Clavicle and AC joint	17	4	43.8
Miscellaneous/basic science	98	24.7	50
Tips and tricks/new techniques	47	11.8	46.8
Total	398	100	45

The overall publication rate was 45.20% after 7 years. Oral presentations were significantly better represented in journals than posters (64.40 vs. 35.40%, *p* < 0.0001). A clear majority of the abstracts that survived the peer-review process (172/180, 95.55%) were published within 4 years of the congress presentation.

**Fig. 1:**
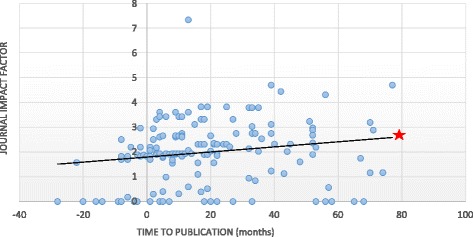
Correlation between time span to publication and impact factor (IF) obtained.  The Spearman correlation between the time to publication and the impact factor of the journal was 0.24 (confidence interval 95% 0.09 to 0.39)

A significant correlation was observed between the number of authors and the publication rates. Abstracts with a greater number of authors (more than three authors) tended to have a better chance of being published (*p* < 0.0001), even for poster presentations (*p* = 0.03). Moreover, 74% of posters with three or fewer authors never achieved subsequent journal publication. The number of authors had no effect on the impact factor obtained in journals for those abstracts that were subsequently published (Spearman’s correlation test 0.06, 95% confidence interval −0.1 to 0.21). The topic of the presentation was not correlated with the probability of publication (*p* = 0.50, Table 1). Abstracts with a longer time to publication in a peer-review journal were published in journals with a higher impact factor (*p* = 0.03). Among those papers that obtained journal publication through a peer-review process, studies published before the SECEC Congress was held exhibited a lower impact factor in journals (1.86) than those published in journals after the congress (2.38, *p* = 0.006) (Fig. 1). Thus, a correlation was found between the impact factor and the time to publication (0.24; Spearman correlation test). However, posters were more represented than oral presentations among papers published beforehand (18 posters, 6 oral presentations, *p* = 0.03). In the adjusted model analysis, the impact factor for abstracts published beforehand continued to be 0.5 points less than those that were published after the congress, despite the influence of the number of posters among those works (*p* = 0.04).

## Discussion

The present study determined that almost half of the podium or poster presentations in the 21st SECEC Congress survived the peer-review process, which is believed to be the gold standard for communicating scientific data. However, the type of presentation (oral and poster presentation) and a greater number of authors listed were related to the possibility of subsequent publication in journals.

The SECEC Congress has become to be an important meeting for shoulder and elbow specialists worldwide. Interest in this European congress is increasing yearly [24]. However, little information is known about the quality of this scientific forum. This congress focuses on the shoulder and elbow subspecialty, but no previous paper has reported this specialty congress publication rate. Only one previous paper reported subspecialty publication rates for the 2001 American Academy of Orthopedic Surgeons Meeting, with a podium and poster publication rate of 53% [23]. The SECEC publication rate is comparable to other international congresses and even to general congresses for trauma and orthopedics [1–3, 6, 7, 13, 15, 18, 23] or specialty meetings [4, 5, 8–12, 14, 16, 17, 19–22] (Table 2).

**Table 2:** Comparison of different congresses publication rates, ordered alphabetically

Congress	Year	GLOBAL PR	PODIUM PR	POSTER PR	Observational period (minimum)
AAHKS [22]	1996–2001	NR	NR	47%	?
AAHKS [14]	1996–2001	NR	58%	NR	?
AAOS [2]	1996	34%			?
AAOS [1]	2001	49%	47%	52%	5 years
AAOS Shoulder and Elbow Sessions [23]	1999–2004	58%	66%	51%	3 years
AANA AOSSM [21]	1990–1993	68.1% 50.9%	NR	NR	?
AOSSM [10]	2006–2010	67.1%	73.3%	56.9%	3 years
EPOS [14]	2006–2008	36.7%	?	?	5 years
German Society Orthopedics and Trauma Surgery [18]	2003	36%	?	?	5 years
ISAKOS [4]	1997 1999	34.6% 39.3%	NR	NR	4 years
OTA [16]	1990–1995	64%	NR	NR	?
OTA [17]	1994–1998		67%	52%	?
POSNA [8]	1991–1994		53%		4 years
SECEC	2008	45.2%	64.4%	35.4%	7 years
Spine Society of Europe [19]	2000–2003	37.8%	48.4	31.3	5 years
Spine Specialty Society (NASS, SRS, ISSLS) [20]	1990–1992 (NASS) 1991–1993 (SRS) 1991–1993 (ISSLS)	40% 47% 45%	NR	NR	4 years

*GLOBAL PR* Global Publication Rate, *PODIUM PR* Podium Publication Rate, *POSTER PR* Poster Publication Rate, *AAHKS* American Academy of Hip and Knee Surgery, *AAOS* American Academy of Orthopedic Surgeons, *AANA* Arthroscopy Association of North America, *AOSSM* American Orthopedic Society of Sports Medicine, *EPOS* European Pediatric Orthopedic Society, *ISAKOS* International Society of Arthroscopy, Knee Surgery & Orthopedic Sports Medicine, *ISSLS* International Society for the Study of Lumbar Spine, *NASS* North American Spine Society, *OTA* Orthopedic Trauma Association, *POSNA* Pediatric Orthopedic Surgery of North America, *SECEC* European Society for Surgery of the Shoulder and the Elbow, *SRS* Scoliosis Research Society

Podium and poster presentations had different publication rates. Poster presentations are commonly thought be published at a lower rate than podium presentations, as podium presentations are typically believed to consist of studies with greater scientific value [17]. Although a significant difference exists between podium and poster presentations, authors presenting poster papers should not feel discouraged by the peer-review publication process because the SECEC poster publication rates are not low.

Previous papers studying different congresses (Orthopedics Research Society Meeting, International Society of Arthroscopy, Knee Surgery and Orthopedic Sports Medicine, American Academy Orthopedic Surgeons Meeting, Australian Orthopedic Association Annual Scientific Meeting, and American Orthopedic Society for Sports Medicine and the Arthroscopy Association of North America Meeting) have suggested that more than 90% of the published abstracts achieve journal publication within 4 years following the congresses [2, 4, 6, 7, 12, 21]. Based on the present paper’s findings, it might be feasible to extend the mean expected time to publication to more than 4 years, as some abstracts (nearly 5%) are published 7 years after congress presentations. This finding could contribute to increasing some congress publication rates, as previous reports have projected the publication of congress abstracts within a shorter period after meetings were held [2, 3, 6, 7, 12, 18].

A new challenge for scientific congress committees is the problem of previously published papers being presented as new abstracts at the meetings. Several papers have reported this problem in various congresses [2, 5, 9, 13, 14, 23]. Preventing plagiarism of their work may explain the authors’ inclination to publish prior to being presented at the congress. However, papers published prior to the congress tend to be published in journals with a lower impact factor, regardless of the type of presentation (abstracts or posters). Moreover, publishing in high impact factor journals may represent a lengthy amount of work for authors, as the delay in publication seems to have a positive effect on the impact factor obtained. The correlation between the time to publication and the impact factor was weak, as coefficient values range from 0.20 to 0.39 in a Spearman’s correlation are typically considered as weak.

The abstract topic had no influence on the journal peer-review process at the 21st SECEC Congress. This finding seems to be different from the findings from other shoulder and elbow congresses, in which the abstract topic plays a role in the probability of publication in a journal [23].

Various reasons have been proposed to explain the disparity between congress presentations and journal publications. A major barrier indicated by authors is the lack of time [26]. This may explain why in the present study, abstracts with a greater number of authors listed were more likely to be published than those with fewer authors. Moreover, most of the unpublished abstracts have never been submitted to the peer-review process [26, 27]. This study had several limitations. One limitation is that this paper analyzed only one SECEC Congress. Furthermore, the reasons for nonpublication were not studied, and the number of abstracts that were not submitted for publication or those that did not survive the peer-review process remains unknown. The strengths of the present study lie in the number of abstracts analyzed, which included both oral presentations and poster abstracts, and the long time to publication.

## Conclusions

In conclusion, less than half of the oral presentations and posters at the 21st SECEC Congress were published in peer-reviewed journals. The SECEC Congress is among the orthopedic specialty meetings with the highest publication rates. Although authors may consider posters as lower-level scientific communications, they have a reasonable publication rate.

## Abbreviations

AAHKS: American Academy of Hip and Knee Surgery
AANA: Arthroscopy Association of North America
AAOS: American Academy of Orthopedic Surgeons
AC: Acromioclavicular
AOSSM: American Orthopedic Society of Sports Medicine
EPOS: European Pediatric Orthopedic Society
GLOBAL PR: Global Publication Rate
ISAKOS: International Society of Arthroscopy, Knee Surgery & Orthopedic Sports Medicine
ISSLS: International Society for the Study of Lumbar Spine
NASS: North American Spine Society
OTA: Orthopedic Trauma Association
PODIUM PR: Podium Publication Rate
POSNA: Pediatric Orthopedic Surgery of North America
POSTER PR: Poster Publication Rate
SECEC: European Society for Surgery of the Shoulder and Elbow
SRS: Scoliosis Research Society
